# Maladaptive blame-related action tendencies are associated with vulnerability to major depressive disorder

**DOI:** 10.1016/j.jpsychires.2021.11.043

**Published:** 2022-01

**Authors:** Suqian Duan, Andrew Lawrence, Lucia Valmaggia, Jorge Moll, Roland Zahn

**Affiliations:** a Institute of Psychiatry, Psychology & Neuroscience, Department of Psychological Medicine, Centre for Affective Disorders, King's College London, London, UK; b Cognitive and Behavioral Neuroscience Unit, D’Or Institute for Research and Education (IDOR), 22280-080, Rio de Janeiro, RJ, Brazil; c South London and Maudsley NHS Trust, London, UK

**Keywords:** Self-blame, Action tendencies, Depression, Moral emotion, Guilt, Shame, Contempt, Indignation, MDD

## Abstract

Biases towards self-blaming emotions, such as self-contempt/disgust, were previously associated with vulnerability to major depressive disorder (MDD). Self-blaming emotions are thought to prompt specific action tendencies (e.g. “feeling like hiding”), which are likely to be more important for psychosocial functioning than the emotions themselves. Systematic investigations, however, of these action tendencies in MDD are lacking. Here, we investigated the role of blame-related action tendencies for MDD vulnerability and their relationship with blame-related emotions. 76 participants with medication-free remitted MDD and 44 healthy control (HC) participants without a history of MDD completed the value-related moral sentiment task, which measured their blame-related emotions during hypothetical social interactions and a novel task to assess their blame-related action tendencies (feeling like hiding, apologising, creating a distance from oneself, attacking oneself, creating a distance from other, attacking other, no action). As predicted, the MDD group showed a maladaptive profile of action tendencies: a higher proneness to feeling like hiding and creating a distance from themselves compared with the HC group. In contrast, feeling like apologising was less common in the MDD than the HC group. Apologising for one's wrongdoing was associated with all self-blaming emotions including shame, guilt, self-contempt/disgust and self-indignation. Hiding was associated with both shame and guilt. Our study shows that MDD vulnerability was associated with specific maladaptive action tendencies which were independent of the type of emotion, thus unveiling novel cognitive markers and neurocognitive treatment targets.

## Highlights

• Blame-related maladaptive action tendencies were distinctive of people with MDD.
• These action tendencies include feeling like hiding and creating a distance from oneself.
• These action tendencies were not predicted by the type of emotion experienced.
• Novel neurocognitive treatments aiming at maladaptive action tendencies are needed.

## 1 Introduction

Previous studies have demonstrated the significance of moral emotions and self-blaming emotional biases as vulnerability factors for major depressive disorder (MDD; (Janoff-Bulman et al., 2009; O’Connor et al., 2002; Power and Dalgleish, 2015; Surguladze et al., 2010). Self-blaming emotions were hypothesized to be associated with action tendencies (Haidt, 2003; Janoff-Bulman et al., 2009; Tangney et al., 2007) which describe a motivational and cognitive state in which there is an increased tendency to engage in certain goal-related behaviours (Haidt, 2003), such as “feeling like hiding”. Determining whether these action tendencies play a role in the vulnerability to MDD is an essential step not only towards a better understanding of psychopathology, but also towards designing novel interventions and risk prediction markers (Lawrence et al., 2021).

Imbalances in blame-related emotions have been shown to be closely related to individuals' negative mental health outcomes and risks of MDD. For example, overgeneralised guilt (O'Connor et al., 2002) and shame have been observed in people with MDD even on remission (Green et al., 2013). In a further study (Zahn et al., 2015a), individuals with remitted MDD exhibited a self-contempt/disgust bias and a reduction in contempt/disgust towards others. These findings demonstrated the potential role of self-blaming emotions as vulnerability traits for MDD that remain present during remission.

Despite the importance of previous findings, existing measures of self-blaming emotional biases have some critical limitations, one of which is the difficulty to distinguish different emotion labels such as shame and guilt, using these measures (Mu and Berenbaum, 2019). In addition, their mechanism in motivating adaptive or maladaptive social actions is elusive. As proposed by Tangney et al. (2007), adaptive moral emotions promote constructive and proactive pursuit, whereas maladaptive moral emotions motivate defensiveness, social withdrawal and interpersonal separation (Tangney et al., 2007). This difference is possibly determined by an individuals’ action tendencies associated with their moral emotions (Haidt, 2003; Tangney et al., 2007). Action tendencies are fundamental characteristics of emotions that closely relate to emotion differentiation and its evolutionary function for the social survival of individuals (Roseman et al., 1994). Previous researchers have proposed associations between moral emotions and action tendencies as well as the adaptive or maladaptive nature of their relationships. For example, although shame and guilt could be either adaptive or maladaptive under different circumstances (Taihara and Malik, 2016), Tangney and colleagues conceived of guilt as being more strongly associated with adaptive action tendencies such as reparative actions including confessions and apologies, whereas they operationalised shame as associated with maladaptive action tendencies such as an attempt to deny, hide, or escape the shame-inducing situation (Haidt, 2003; Janoff-Bulman et al., 2009; Tangney et al., 2007).

Indeed, the nature of action tendencies (adaptive vs. maladaptive) might be important in guiding the behavioural consequences of moral emotions, thereby providing a direct link to vulnerability to psychopathology (O'Connor et al., 1997; Tangney et al., 2007). Of particular relevance in MDD is the action tendency related to self-blaming emotions such as self-contempt and shame. Consistent with this idea, a previous study suggests that action tendencies associated with self-blaming emotions (e.g. withdrawal) predicted higher depressive symptoms in undergraduate students, although they did not differentiate specific types of withdrawal related to depression (Mu and Berenbaum, 2019).

Here, we focus on two types of withdrawal-related maladaptive action tendencies that potentially contribute to MDD vulnerability: hiding and creating a distance from oneself. Feeling like hiding has been entailed in the operationalizations of self-blaming feelings such as shame (Tangney et al., 2007) shown to be associated with depressive symptoms, but their systematic investigation in clinical depression is lacking. Whilst the action tendency of creating a distance from others has been well described in relation to disgust and we have previously found self-disgust to be increased in MDD (Zahn et al., 2015a), the associated action tendency of creating a distance from oneself has not been investigated in depression. Some of our patients have described this as wanting to escape their body and feelings. This is similar to the feeling of depersonalisation found in classical descriptions of depression (Kendler, 2017), which has been classified as a disturbance of the ego perception in its identity or unity (Faehndrich and Stieglitz, 1997). However, depersonalisation is an involuntary sensory experience rather than a feeling of wanting to experience it. These dissociation-related phenomena in depression have been conceptualised as a form of cognitive avoidance (Holmes et al., 2016; Lemogne et al., 2006). Indeed, individuals with MDD had more escape/avoidance coping styles (Haskell et al., 2020), which might be associated with an action tendency to hide, or create a distance from themselves in the first place. In contrast, action tendencies such as repair and apologising, were found to predict lower depressive symptoms in a non-clinical sample (Mu and Berenbaum, 2019), which shows their possible adaptive roles in the vulnerability to MDD.

Despite the potential distinctive psychopathology of feeling like creating a distance from oneself and feeling like hiding, to our knowledge, these action tendencies have so far not been directly assessed in individuals with MDD. Furthermore, there is no systematic investigation of how action tendencies and moral emotions are linked in MDD. The present study aimed to elucidate these questions by directly examining the relationship between blame-related emotions and action tendencies, and their potential role in MDD vulnerability. To identify potential vulnerability traits associated with MDD (Bhagwagar and Cowen, 2008), understanding the differences between remitted MDD and healthy control groups is a first step, as the risk of depressive episodes MDD even after a single episode (50%) is far higher than in people with no personal history (15%) (Eaton et al., 2008). We developed a novel action tendency task that specifically assessed different action tendencies (feeling like: apologising, hiding, creating a distance from oneself, attacking oneself) when people experienced self-blame-related emotions (shame, guilt, self-contempt/disgust, self-directed anger) and comparing them against emotions and action tendencies associated with blaming others. We hypothesized that individuals with fully remitted MDD were more likely to have maladaptive action tendencies reflecting their vulnerability to further episodes despite current symptom remission, when compared with a control group without a personal and family history of MDD. More specifically, we firstly hypothesized that individuals with MDD have an increased maladaptive action tendency to create a distance from themselves and/or to hide. Secondly, based on the distinctive role of self-contempt and shame in MDD (Zahn et al., 2015a), we hypothesized that self-contempt/disgust is associated with feeling like creating a distance from oneself and thirdly, that shame is distinctively associated with feeling like hiding. In addition, as overgeneralised forms of self-blame is one central feature of MDD (O'Connor et al., 1997), we also explored whether there is an overgeneralised perception of control and responsibility of action tendencies in the MDD group.

## 2 Materials and methods

### 2.1 Participants

Seventy-six medication-free participants with remitted MDD and 44 healthy control (HC) participants took part in the study and completed both value-related moral sentiment (VMST) and action tendency task. Participants were recruited via online and print advertisements as part of a bigger project and results of the VMST and psychopathological characteristics have been previously reported (Zahn et al., 2015b). A total of 707 people took part in an initial phone screening interview to establish whether they would be invited to a clinical assessment using the Structured Clinical Interview-I for DSM-IV (First et al., 1997). The inclusion criteria were a diagnosis of MDD and a remission period for at least six months for the MDD group, and no history of an axis-I disorder or first-degree relatives with mood disorders or schizophrenia for the HC group. The details of the exclusion and inclusion process of participants were included in the supplementary materials (See Supplementary Table 1).

Following the face-to-face assessment, 76 participants with MDD and 44 HC participants met all the criteria and took part in the current study. Demographic information and clinical characteristics of the included participants are shown in Table 1 and Supplementary Table 2, respectively. There were no significant differences regarding the age, sex, nor the years of education of the two groups. As to be expected, depressive symptoms of MDD participants were slightly but significantly higher than those of HC participants as measured by the Beck Depression Inventory (Beck et al., 1961) total score and the Montgomery-Asberg Depression Rating Scale (Montgomery and Åsberg, 1977) total score.

**Table 1:** Demographic characteristics of participants.

	Sample		
	rMDD (n = 76)	HC (n = 44)	p-value
Age	35.90 (13.10)	33.70 (12.90)	0.38
Sex*[Female]	55 (72.40%)	28 (63.60%)	0.43
Years of Education	16.80 (2.16)	17.30 (2.48)	0.25
BDI Score	3.71 (3.73)	0.84 (1.63)	<0.001
MADRS Score	1.08 (1.49)	0.59 (1.19)	0.05
GAF Score	85.30 (5.87)	89.10 (2.60)	<0.001

BDI = Beck Depression Inventory; MADRS = Montgomery-Asberg Depression Rating Scale; GAF - Global Assessment of Function. Values are Mean (Standard Deviation) for approximately continuous variables and Count (Percentage%) for categorical variables. Summary p-values are obtained from Welch's t-tests and Fisher's exact test respectively.

### Ethical approval

This study was approved by the South Manchester NHS Research Ethics Committee. All participants have given written informed consent after the procedures of the study have been fully explained.

### 2.2 Assessment of blaming emotions and action tendencies

Participants were asked to complete two tasks for assessing their blame-related emotions and blame-related action tendencies respectively. Both was completed at home using excel macros, following their baseline assessments.

Participants' blame-related emotions were assessed using the value-related moral sentiment task (VMST), which has been described and validated in our previous studies (Green et al., 2012; Lythe et al., 2015). At the beginning of the VMST, participants were asked to enter the name of their best friend. Then they were presented with sentences containing hypothetical social interactions in which either the participant (in the self-agency condition) or their best friend (in the other-agency condition) acts contrary to social and moral values [e.g. you act bossily towards (the name of the participant's friend)]. The same social interactions were used for both self-agency and other-agency conditions, with 90 trials in each condition. 50% of the trials used negative social behaviours (e.g., does act bossily) and 50% used negated positive social behaviours (e.g., does not act bossily). For each trial, participants were asked to rate the unpleasantness of each social interaction using a 1–7 point Likert scale, where 1 indicates not unpleasant at all and 7 indicates extremely unpleasant. Valid trials were defined as those that were perceived as highly unpleasant (those rated at the individual median or above in the VMST). They were also required to choose the feeling that they would feel most strongly from four self-blaming emotions (shame, guilt, contempt/disgust towards self, and indignation/anger towards self) and two other-blaming emotions (contempt/disgust towards friend or indignation/anger towards friend) as well as no/other emotions. One moral emotion was chosen for each trial and 180 moral emotions per participant were chosen in the VMST in total.

In the novel action tendency task, all the hypothetical social interactions (180 trials) in the VSMT were shown again. Participants were instructed to select one action that they would most strongly feel like doing from eight different action tendencies (feel like verbally or physically attacking/punishing your friend, feel like verbally attacking or physically attacking/punishing yourself, feel like apologising/fixing what you have done, feel like hiding, feel like creating distance from your best friend, feel like creating distance from yourself, no action, other action). Participants were also asked to rate how responsible they would feel, and how much control they felt they would have for each social action using a 1–7 point Likert scale, where 1 indicated “not at all”, and 7 indicated “extremely/completely”. In total, 180 action tendencies for 180 trials were chosen for the action tendency task. The proportions of choosing each action tendency across all valid trials was computed for each participant. The split-half reliability coefficients of the action tendency task were high (>0.79) for each action tendency measure in each agency condition (see Supplementary Table 3). Supplementary Fig. 1 shows a screenshot of self-agency condition of one social interaction in the action tendency task, in which the participant entered their best friend's name at the beginning.

### 2.3 Data analysis

All statistical analyses in the study were carried out using R software. A complete case approach was taken for each planned analysis. To test our first hypothesis (individuals with MDD had an increased tendency to create a distance towards themselves and/or hide), a repeated measures MANOVA was conducted to examine whether the proportion of trials selected by participants differed by action tendency, agency condition (self-vs. other), and clinical group and whether there were interactions between the three variables. Post-hoc tests for between-group differences in each action tendency over both agencies were conducted. Multiple comparison corrections were carried out for all post-hoc tests.

To test our second and third hypotheses (self-contempt/disgust was associated with feeling like creating a distance from oneself and shame was associated with feeling like hiding), the relationships between moral emotions and each action tendency were tested. Six mixed effect logistic regression models were conducted for each action tendency as outcome variable, with its related moral emotions, group (MDD vs. HC) as well as their interactions as predictors. More specifically, apologising, hiding, creating a distance from oneself, and attacking oneself are likely to be motivated by self-blaming emotions (shame, guilt, self-disgust/contempt, and self-indignation). Therefore, in Model 1, 2, 3 and 4, we used self-blaming emotions, group and their interactions to predict apologising, hiding, creating a distance from oneself and attacking oneself. Creating a distance from one's friend and attacking one's friend are likely to be motivated by other-blaming emotions. Therefore, in Model 5 and 6, we used other-blaming emotions, group, and their interactions to predict creating a distance from one's friend and attacking one's friend. In all trials, moral emotions and action tendencies were coded as either 1 or 0 (1 for yes and 0 for no). Reference categories for moral emotions were the trials in which participants chose no/other emotion.

Participants were excluded if they chose more than one moral emotion or action tendency in more than 5% of the trials (9 trials). This ensured that all participants included in the logistic regression models understood the instruction of the tasks and could distinguish different moral emotions and action tendencies. In addition, only valid trials were included (trials that were perceived highly unpleasant and rated at the individual median or above). The significance threshold was set to an approximate Bonferroni-corrected p < .05 across all 6 models, corresponding to an uncorrected p < .008 in each model.

Perception of control in the hypothetical social scenarios were compared between two groups using Welch's t-tests. In addition, to exclude possible role of scaring effect of previous depressive episodes, Kendall's rank correlations were used to test the correlation between proportions of choosing each maladaptive action tendency, number of previous depressive episodes, and participants' BDI scores.

## 3 Results

### 3.1 Group differences for choosing different action tendencies

Proportion of trials for each action tendency and each agency is presented in Table 2. We observed group differences that were action tendency- and agency-specific (see Table 3). Post-hoc tests for between-group differences in each action tendency over both conditions are presented in Table 4. Our first hypothesis was confirmed that MDD patients more frequently felt like hiding than control participants in both conditions and that there was a significantly higher proportion of feeling like creating a distance from oneself in the self-agency condition for MDD participants. There was a lower proportion of feeling like apologising for the MDD group compared with the control group in the self-agency condition. In contrast, participants with MDD were more likely to feel like apologising in the other-agency condition. MDD group also had a higher proportion of feeling like attacking oneself in the self-agency condition, but not in the other-agency condition (see Table 4 for details). In addition, participants with MDD had a significantly higher perceived control in the other-agency conditions relative to HC participants (Welch's t = −0.52, p = 0.003) which drove the group difference in the measure of overgeneralised perception of control which we defined as the difference score between control in the self- and other-agency condition (Welch's t = 0.56, p = 0.01; also see Supplementary Fig. 2).

**Table 2:** Means and standard deviations of proportion of trials for which a particular action tendency was chosen.

Action	Healthy Control (n = 44)		MDD (n = 76)	
	self-agency	other-agency	self-agency	other-agency
No action	0.36 (0.23)	0.57 (0.28)	0.32 (0.21)	0.47 (0.26)
Apologise	0.53 (0.23)	0.05 (0.07)	0.45 (0.22)	0.09 (0.12)
Distance from friend	0.03 (0.05)	0.30 (0.23)	0.05 (0.06)	0.32 (0.23)
Distance from self	0.02 (0.06)	0.01 (0.02)	0.06 (0.08)	0.01 (0.03)
Hide	0.03 (0.05)	0.01 (0.02)	0.06 (0.09)	0.05 (0.11)
Attack friend	0.001 (0.004)	0.05 (0.12)	0.002 (0.01)	0.05 (0.10)
Attack self	0.03 (0.11)	0.02 (0.05)	0.07 (0.14)	0.01 (0.05)

**Table 3:** Action tendencies by group and condition.

Effect	df	F-value	p-value
group	1, 118	8.95	0.003**
action tendency	2.7, 323.3	206.45	<.001***
group x action tendency	2.7, 323.3	2.59	0.06
agency	1, 118	0.10	0.75
group x agency	1, 118	5.35	0.02*
action tendency x agency	3.7, 435.4	189.45	<0.001***
group x action tendency x agency	3.7, 435.4	4.16	0.003**

The proportion of trials for which a particular action tendency was selected was arcsine square root transformed for this repeated-measures multivariate analysis of variance (MANOVA). Repeated measures MANOVA revealed a significant omnibus interaction: action tendency x agency x clinical group, F(3.69, 435.37) = 4.16, p = .003. ***p<0.001, **p<0.01, *p<.05.

**Table 4:** Action tendencies by group and condition - post-hoc tests.

agency	action tendency	estimate	t-value	p-value
self-agency	no action	0.05	1.14	0.25
	apologise	0.09	2.15	0.03*
	create a distance from other	−0.04	−0.91	0.36
	create a distance from oneself	−0.09	−2.27	0.02*
	hide	−0.08	−1.99	0.05*
	other-attack	−0.01	−0.24	0.81
	self-attack	−0.09	−2.36	0.02*
other-agency	no action	0.13	3.43	<0.001***
	apologise	−0.08	−2.15	0.03*
	create a distance from other	−0.03	−0.76	0.45
	create a distance from oneself	−0.02	−0.52	0.60
	hide	−0.10	−2.51	0.01*
	other-attack	−0.01	−0.20	0.84
	self-attack	0.00	0.07	0.94

Estimated effect of MDD group: HC mean - MDD mean. ***p<0.001, **p<0.01, *p <0.05.

### 3.2 The relationship between self-blaming emotions and action tendencies

As shown in Table 5 and Supplementary Fig. 3, all self-blaming emotions were associated with a higher probability of apologising across groups. Shame and guilt were both associated with a higher probability of hiding. Interestingly, self-indignation anger rather than self-disgust/contempt was associated with a higher probability of creating a distance from oneself across groups. Reversely self-disgust/contempt correlated with a higher probability of feeling like attacking oneself rather than creating a distance from oneself. No other main effect of group or group by emotion interactions were found for any of the other self-blame-related action tendencies (apologising, hiding, attacking oneself).

**Table 5:** Self-blame-related action tendencies and emotions.

	Apologising vs. Reference category			Hiding vs. Reference category			Distancing from self vs. Reference category			Attack self vs. Reference category		
	**B**	**SE**	**p**	**B**	**SE**	**p**	**B**	**SE**	**p**	**B**	**SE**	**p**
**Shame**	1.96	0.22	<0.001***	3.67	0.84	<0.001***	1.38	0.77	0.07	2.31	1.18	0.05
**Guilt**	1.93	0.18	<0.001***	2.32	0.78	0.002**	1.20	0.63	0.06	2.06	1.10	0.06
**Self-disgust/contempt**	1.83	0.23	<0.001***	2.03	0.91	0.03	0.92	0.70	0.19	3.20	1.17	0.006**
**Self-anger**	2.00	0.23	<0.001***	1.33	0.75	0.03	2.67	0.68	<0.001***	1.89	.1.52	0.13
**Group**	0.24	0.27	0.38	1.63	0.85	0.02	2.21	1.03	0.03	2.29	1.49	0.12
Group*Shame	-0.71	0.28	0.01	−1.38	0.81	0.03	-0.27	0.88	0.76	0.65	1.36	0.63
Group*Guilt	-0.40	0.23	0.09	-0.80	0.77	0.11	-0.70	0.75	0.34	0.65	1.27	0.61
Group*Self-disgust/contempt	-0.34	0.30	0.24	0.03	0.89	0.84	-0.71	0.82	0.39	0.01	1.35	0.99
Group*Self-Anger	-0.37	0.31	0.23	0.005	0.89	0.79	−1.50	0.87	0.08	1.20	1.47	0.41

Four mixed effect logistic regression models were conducted, one for each self-blame-related action tendency as outcome variable. Predictors were agency-congruent moral emotions (Shame, guilt, self-disgust/contempt, self-anger), group (MDD vs. HC) as well as their interactions. Reference categories for moral emotions in all models were the trials in which participants chose no/other emotion. The significance threshold was set to an approximate Bonferroni-corrected p < 0.05 across all 6 models, corresponding to an uncorrected p < 0.008 in each model. ***p<0.001, **p<0.01. B = estimate, SE = standard error.

### 3.3 The relationship between other-blaming emotions and action tendencies

Table 6 and Supplementary Fig. 3 show that the other-blaming emotions including contempt/disgust and anger/indignation towards others were both associated with a higher probability of attacking others and distancing from others across both groups. No main effect of group nor interaction between group and other-blaming emotions were found. The relationship of clinical variables with maladaptive action tendencies in MDD was presented in the supplementary materials.

**Table 6:** Other-blame-related action tendencies and emotions.

	Attacking friend vs. Reference categories			Distancing from friend vs. Reference categories		
	**B**	**SE**	**p**	**B**	**SE**	**p**
Disgust/Contempt towards friend	2.39	0.58	<0.001***	1.70	0.20	<0.001***
Anger/Indignation towards friend	2.76	0.46	<0.001***	1.46	0.14	<0.001***
Group	1.03	0.86	0.23	0.61	0.36	0.09
Group * Disgust/contempt towards friend	-0.50	0.73	0.49	-0.46	0.26	0.08
Group * Anger/indignation towards friend	0.26	0.58	0.65	0.03	0.19	0.87

Two mixed effect logistic regression models were conducted, one for each other-blame-related action tendency as outcome variable. Predictors were agency-congruent moral emotions (disgust/contempt towards friend, anger/indignation towards friend), group (MDD vs. HC) as well as their interactions. Reference categories for moral emotions in all models were the trials in which participants chose no/other emotion. The significance threshold was set to an approximate Bonferroni-corrected p < 0.05 across all 6 models, corresponding to an uncorrected p < 0.008 in each model. ***p<0.001, *p<0.01. B = estimate, SE = standard error.

## 4 Discussion

We hypothesized that individuals with MDD were more likely to experience maladaptive self-blame-related action tendencies which interfere with reparative actions. We confirmed our first specific hypothesis that individuals with MDD would display an increased tendency to feel like creating a distance from themselves and hiding. Our third specific hypothesis was supported by finding that shame was associated with feeling like hiding, in line with what has been proposed in the literature (Roseman et al., 1994; Tangney et al., 2007). However, contrary to our second hypothesis, self-contempt/disgust was associated with attacking oneself rather than feeling like creating a distance from oneself. Intriguing was the finding of an overgeneralised perception of control for other people's actions and a tendency to apologise for others' wrongdoings in the MDD group.

Previous research has demonstrated the distinction between adaptive and maladaptive action tendencies. The former promotes constructive and proactive pursuit, such as apologising, whereas the latter motivates defensiveness, social withdrawal and interpersonal separation, such as hiding and creating a distance from oneself (Tangney et al., 2007). Our study is the first to demonstrate that maladaptive action tendencies distinguish participants with MDD and healthy control participants, who were closely matched apart from their difference in MDD vulnerability. Despite a lack empirical studies in clinical populations, this finding is consistent with Mu and Berenbaum (2019), who also demonstrated that withdrawal-related maladaptive action tendencies are associated with vulnerability to depression in a non-clinical sample. Maladaptive action tendencies as demonstrated here could contribute to maladaptive coping styles, such as avoidance-oriented coping, which predicted anxious and fearful responding under stressful circumstances (Spira et al., 2004) and have been consistently found to be associated with MDD (Berghuis and Stanton, 2002; Burker et al., 2005). Indeed, creating a distance from oneself and hiding might prepare people to use social avoidance as a coping mechanism, which contribute to their MDD vulnerability in response to negative social feedback. This view is consistent with Lemogne and colleagues (Lemogne et al., 2006), who suggest that patients with MDD tend to recall memories from a third person perspective and use it as a form of cognitive avoidance. In addition, from a more theoretical perspective, feeling like creating a distance from oneself might also involve the rejection and denial of one's self-identity, as well as an increased self-discrepancy as proposed by Higgins (1987). An increase in self-discrepancy was both directly and indirectly linked to depressive symptoms (Roelofs et al., 2007) and possibly also related to one's vulnerability to MDD.

In addition, the finding that people with MDD tend to apologise more readily when their friend has done something wrong is consistent with our previous finding that individuals with MDD have increased overgeneralised self-blaming emotions and reduced blame-related emotions towards others (Zahn, Lythe et al., 2015). The overgeneralised self-blame in MDD is supported by the attributional theory of depression (Abramson et al., 1978), which states that people with depression tend to attribute negative consequences to internal, stable and global rather than external factors. Indeed, when experiencing stressful events, people with depression might blame themselves even though they have not done anything wrong, as seen in our findings. As proposed by Abramson and colleagues, these kinds of negative attributional styles were hypothesized to reduce individuals' self-esteem and ultimately increase their vulnerability to depression (Abramson et al., 1978). We also found that people with MDD tend to attribute control to themselves and this overgeneralised control over other people's wrongdoing could also be a vulnerability trait of MDD which is in keeping with increased omnipotent responsibility guilt in MDD (O'Connor et al., 2002).

On the other hand, our findings are inconsistent with the view that shame is specifically associated with maladaptive action tendencies that promote defensiveness, interpersonal separation and distance, and guilt is specifically associated with adaptive action tendencies that motivate constructive and reparative actions (Ketelaar and Tung Au, 2003; Tangney et al., 2007). Instead, all self-blaming emotions were associated with a tendency to apologise, but that guilt and shame can also both be associated with feeling like hiding. These differences indicate that shame and guilt can be either adaptive or maladaptive under different circumstances (Taihara and Malik, 2016) and their adaptive or maladaptive forms cannot be distinguished based on the emotion labels alone. Our findings therefore reveal the limitations of measuring self-blaming emotions using emotion labels and imply that action tendencies could be a more appropriate measure to assess the adaptiveness of self-blame (Mu and Berenbaum, 2019).

In addition, an observed association between self-contempt/disgust and feeling like attacking oneself is also at odds with our hypothesis and the hypotheses proposed by previous researchers which state that contempt/disgust is specifically associated with creating a distance (Haidt, 2003). However, most previous hypotheses concern contempt towards others, not oneself, which might suggest that these two emotions are not comparable and motivate very different action tendencies. Interestingly, the unexpected association between self-contempt and self-attacking helps to explain why depressed individuals who have biases towards self-contempt also had a high rate of self-harm (Duan et al., 2020, Stanicke, 2021). That is, the proneness to self-contempt in depressed individuals motivates a tendency to attack themselves when stressful events occur, which then promotes their self-harming behaviours. Nevertheless, this hypothesized relationship needs to be further examined.

### 4.1 Limitations

On a more cautionary note, our study was limited firstly by its cross-sectional design, which made it difficult to infer a causal relationship between MDD vulnerability and action tendencies. While maladaptive action tendencies could be a vulnerability trait for MDD, it is also possible that these represent scarring effects of previous depressive episodes (Wichers et al., 2010). Nevertheless, this is unlikely as no correlation was found between maladaptive action tendencies and the number of previous depressive episodes. Future studies are needed to determine whether feeling like creating a distance from oneself is a pure trait marker of vulnerability or is also modulated by depressive state which its correlation with residual symptoms suggests. Secondly, the task was in a verbal format and included abstract descriptions of scenarios. Thus, participants' self-blaming emotions might have depended in part on how well they can imagine the scenarios and indeed we previously found an association between visual imagery ratings and emotional intensity ratings (Zahn et al., 2009). It is therefore important to develop more immersive and ecologically valid tasks to measure moral emotions and blame-related action tendencies in future studies, which rely less on the ability to create one's own imagery, which recent research shows is a widely varying ability (FeldmanHall et al., 2012; Fulford et al., 2018).

## 5 Conclusions

Taken together, feeling like creating a distance from oneself and hiding were distinctive for remitted MDD compared with the control group, thus unveiling a novel marker of psychopathology, which was present even when symptoms had subsided. Future studies are needed to probe the prognostic value of maladaptive action tendencies. If replicated, our findings can guide the development of novel psychological and neurocognitive treatments specifically aiming at self-distancing and hiding which are so far neither assessed nor addressed in standard psychotherapeutic approaches.

## Funding

This study was funded by an 10.13039/501100000265MRC Clinician Scientist Fellowship to RZ (G0902304). RZ was partly funded by the 10.13039/501100000272National Institute for Health Research (10.13039/501100000272NIHR) 10.13039/100014461Biomedical Research Centre at 10.13039/100009362South London and Maudsley NHS Foundation Trust and King's College London and by a 10.13039/100009670NARSAD Independent Investigator Grant (24715) from the 10.13039/100000874Brain & Behavior Research Foundation. SD was partly funded by a Henry Lester Trust Award. This study was funded by the UK 10.13039/501100000265Medical Research Council (G0902304).

## Author contributions

Study design: SD, AL, LV, JM, RZ.

Data collection: RZ.

Data analysis, and interpretation of data: SD, AL, RZ.

Manuscript preparation: SD, AL.

## Declaration of competing interest

RZ is a private psychiatrist service provider at The London Depression Institute, has collaborated with e-health companies EMIS PLC and Alloc Modulo Ltd. He has received honoraria from pharmaceutical companies (Lundbeck, Janssen) for scientific presentations and is a co-investigator for a Livanova-funded study on Vagus Nerve Stimulation. None of the other authors report biomedical financial interests or potential conflicts of interest related to the subject of this paper.
