# Supplementary material for: Maladaptive blame-related action tendencies are associated with vulnerability to major depressive disorder
Source: J Psychiatr Res. 2022 Jan;145:70–6. doi: 10.1016/j.jpsychires.2021.11.043 (PMC8756141; doi:10.1016/j.jpsychires.2021.11.043)
Supplement: Multimedia component 1 [file mmc1.docx]

**Supplementary materials**

**Maladaptive blame-related action tendencies are associated with vulnerability to major depressive disorder**

Suqian Duan^1^, Andrew Lawrence^1^, Lucia Valmaggia^1^, Jorge Moll^2^, Roland Zahn^1,2,3*^

*^1^ Institute of Psychiatry, Psychology & Neuroscience, Department of Psychological Medicine, Centre for Affective Disorders, King’s College London, London, SE5 8AZ, UK*

*^2^ Cognitive and Behavioral Neuroscience Unit, D’Or Institute for Research and Education (IDOR), 22280-080 - Rio de Janeiro, RJ, Brazil*

*3 South London and Maudsley NHS Trust, London, UK*

*Corresponding author

Dr Roland Zahn (see address above)

E-mail: roland.zahn@kcl.ac.uk

Phone: 0044-(0)20 7848 0348

Fax: 0044-(0)20 7848 0298

**Supplementary Methods**

*Inclusion and exclusion of participants*

A total of 707 people took part in an initial phone screening interview to establish whether they would be invited to a clinical assessment using the Structured Clinical Interview-I for DSM-IV (First, Gibbon, Spitzer, Benjamin, & Williams, 1997). The inclusion criteria were a diagnosis of MDD and a remission period for at least six months for the MDD group, and no history of an axis-I disorder or first-degree relatives with mood disorders or schizophrenia for the HC group. After the initial phone screening interview, 276 people passed the screening with 184 in the MDD group and 92 in the HC group (431 people were excluded at this stage, the exclusion reasons following the phone screening interview are listed in Supplementary Table 1). Participants were then invited to see a senior psychiatrist (RZ) and take part in a face-to-face clinical assessment to further exclude the possibility of current co-morbid axis-I and relevant past axis-I disorders (full inclusion and exclusion criteria and assessment details can be found in Zahn, Lythe, Gethin, Green, Deakin, Workman, et al. (2015). Following the face-to-face assessment, 76 participants with MDD and 44 HC participants met all the criteria and took part in the current study.

**Supplementary Results**

*Proportion of trials for each action tendency*

Means and standard deviations of proportion of trials for each action tendency are presented in Supplementary Table 4*.* Over both groups clear differences in action tendency selection were seen between the self-agency and other-agency conditions, as expected. Feeling like attacking (self- or other-) was highly agency-specific with agency-incongruent options (e.g. feeling like attacking other in the self-agency condition) occurring rarely. In self-agency trials, apologising, creating a distance from self and attacking self were more common. In the other-agency condition, distancing from one’s friend and feeling like attacking one’s friend were more common. In contrast to feeling like creating a distance from oneself, feeling like hiding did not differ between conditions.

*Relationship of clinical variables with maladaptive action tendencies in MDD*

There were no significant correlations between proneness to feeling like hiding, or creating a distance from oneself and the number of previous depressive episodes (τ=-.13 , p=.15, z=-1.46; τ=.07, p=.42, z=-.80). BDI scores were weakly positively correlated with proneness to feeling like creating a distance from oneself (τ=.31, p<.001, z=3.47), but not with feeling like hiding (τ=.14, p=.12, z=1.56).

**Supplementary Table 1| Exclusion reasons for participants following phone pre-screening interview.**

| Reason for exclusion | n |
| --- | --- |
| Current antihypertensive medications or statins | 20 |
| Current antidepressant or other centrally active medications | 52 |
| Diabetes | 4 |
| Epilepsy | 5 |
| Multiple sclerosis | 3 |
| Past cancer | 7 |
| Past stroke | 1 |
| Thyroid function problems | 19 |
| Vitamin D deficiency | 1 |
| Other psychiatric disorders than MDD | 54 |
| Substance or alcohol abuse | 23 |
| Other general medical condition | 5 |
| Family history of MDD/bipolar/schizophrenia (control group) | 26 |
| Excluded because of age-matching (control group) | 3 |
| Left-handed | 20 |
| MRI contraindications | 77 |
| Non-native English speaker | 19 |
| Out of age range | 4 |
| No reason recorded | 5 |
| Withdrawal after phone pre-screening | 33 |
| Not meeting full screening criteria for MDD | 30 |
| Not remitted for long enough | 7 |
| Fulfilling criteria for current MDD | 13 |
| Total excluded after phone pre-screening | 431 |

**Supplementary Table 2 | Clinical characteristics of included MDD participants (n=76)**

| *Mean age at onset (Years)* | 22.74 ± 9.96 (range:11-52) |
| --- | --- |
| *Past MDD subtype* |  |
| With melancholic feature | 42 |
| With atypical feature | 8 |
| No specific subtype | 26 |
| *Number of previous MDEs* |  |
| 1 | 17 |
| 2 | 22 |
| 3 | 14 |
| 4 | 9 |
| 5 or more | 14 |
| Average number of previous MDEs | 3.84 ± 6.39 (range:1-53) |
| *Last MDE details* |  |
| Average length of MDE (months) | 13.29 ± 17.49 (range:.5-96) |
| Average time in remission (months) | 28.85 ± 37.30 (range:5.5-282.0) |
| Total illness duration | 112.11 ± 107.71 (range:2-552) |
| *Life-time axis-I co-morbidity* ^a^ |  |
| Eating disorder | 2 |
| Post-traumatic stress disorder | 2 |
| No life-time co-morbidity | 56 |
| Unknown | 14 |
| *Family history* |  |
| Fist degree relative with MDD | 27 |
| No family member with history of  MDD | 26 |
| First degree relative with  schizophrenia or bipolar disorder | 6 |
| Unknown or diagnostically unclear | 15 |

MDE: major depressive episode; MDD: major depressive disorder; MDD subtype classification was based on adapting the SCID-I for DSM-IV-TR to allow lifetime assessment of subtypes. a. All co-morbid disorders were fully remitted at time of study and none of the co-morbid disorders was a likely primary cause of the depressive episodes.

All ± refer to standard deviations.

**Supplementary Table 3| Split-half reliability of the action tendency task**

| Action tendency task response | Self-agency condition | Other-agency condition |  |
| --- | --- | --- | --- |
| Apologising [%] | .89 | .90 |  |
| Hiding [%] | .86 | .93 |  |
| Attacking self [%] | .95 | .87 |  |
| Creating a distance from oneself [%] | .94 | .79 |  |
| Attacking friend [%] | .84 | .93 |  |
| Creating a distance from friend [%] | .86 | .93 |  |
| Perceived control [mean] | .96 | .96 |  |
| Perceived responsibility [mean] | .96 | .98 |  |

Split-half reliability was calculated for each action tendency response per condition using the Spearman-Brown

formula after randomly splitting items into parallel forms based on the alphabetic order of stimuli.


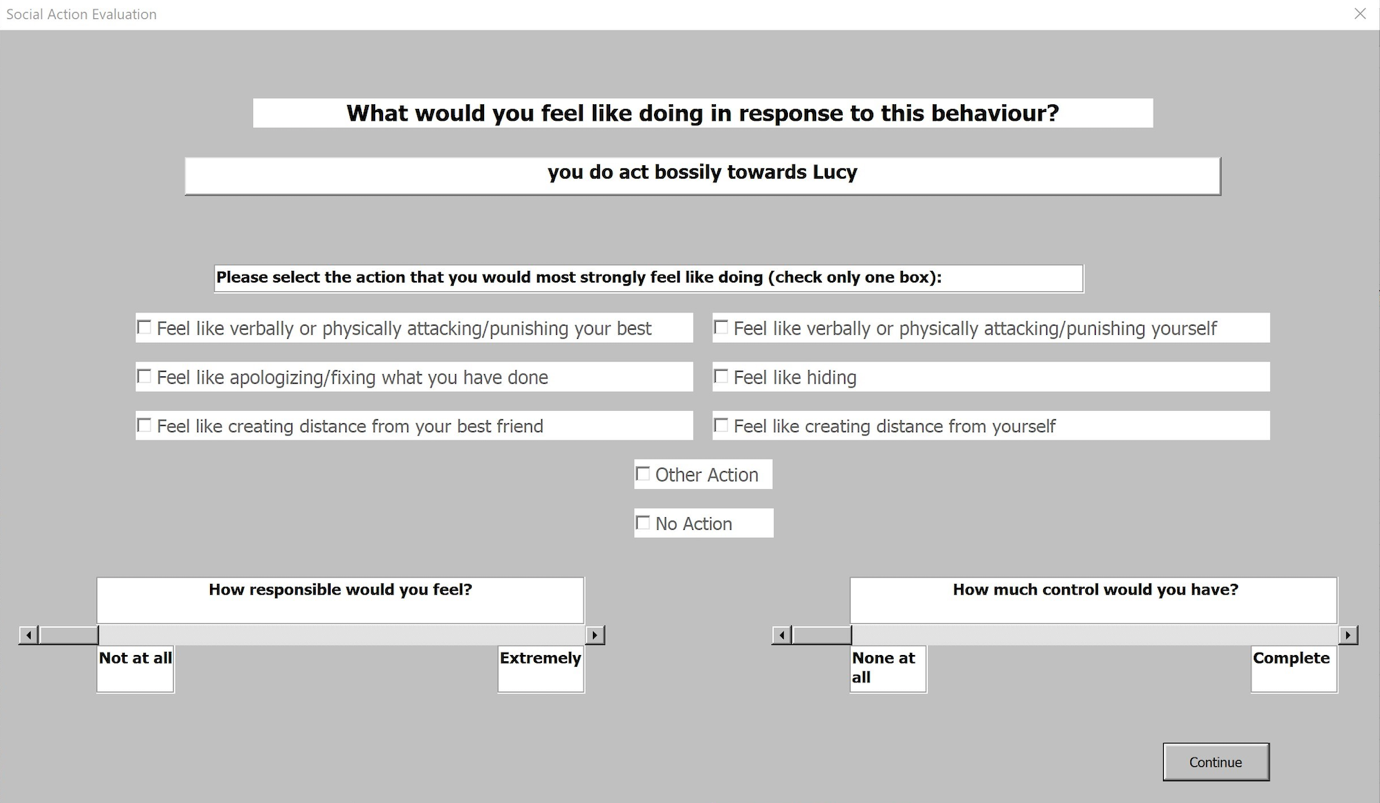


**Supplementary Figure 1|** A trial in the self-agency condition of the action tendency task that shows a hypothetical social interaction between the participant and her best friend (The participant does act bossily towards her best friend, Lucy). There are 180 trials in total, with 90 of them in the self-agency and other-agency condition respectively. Participants were asked to choose between eight action tendencies as well as how responsible they felt and how much control they had for all trials. Everything will stay the same in the other-agency condition, except that the scenario will change to “Lucy does act bossily towards you”.


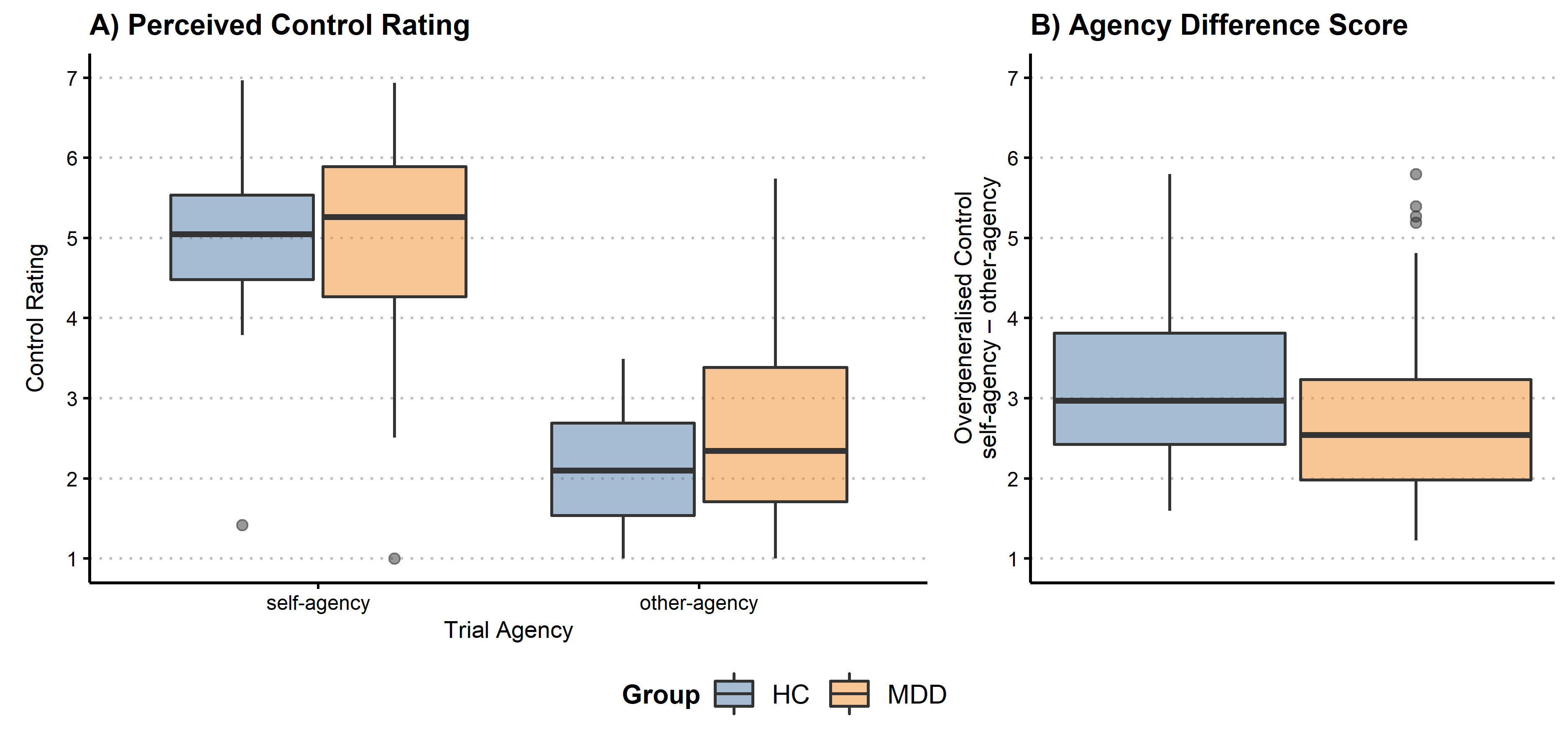


**Supplementary Figure 2|** Perception of control (A) and the level of overgeneralised control (B) by clinical groups with 95% confidence intervals as measured by the valued-based moral sentiment task. Overgeneralised control was calculated by the other-agency control rating subtracted from the self-agency control rating for each subject. A higher value indicates less overgeneralisation of control ratings.


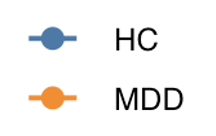
**
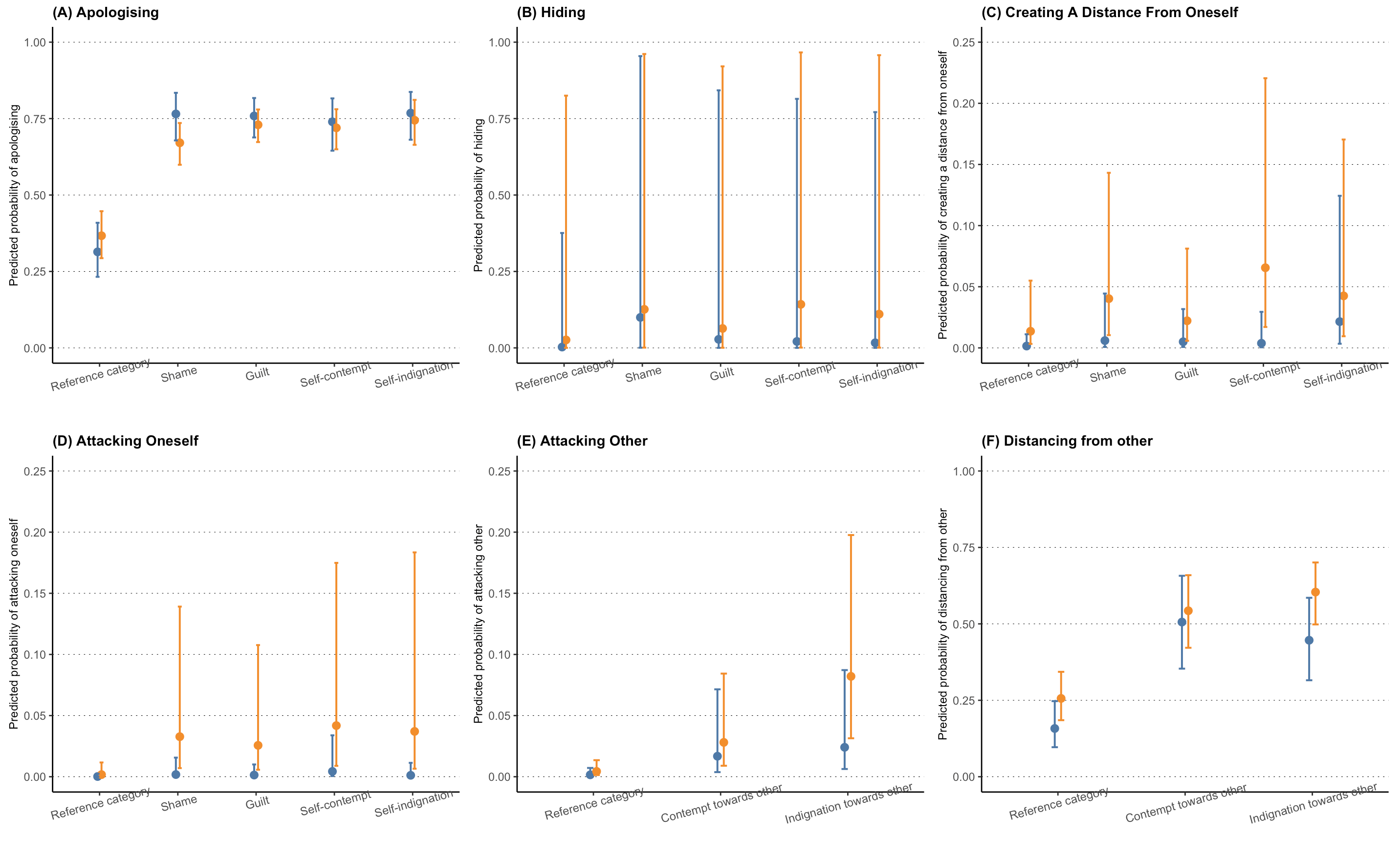
**

**Supplementary Figure 3|** Displayed are the means and 95% confidence intervals for the predicted probability of feeling like apologising (A), hiding (B), creating a distance from oneself (C), attacking oneself (D), attacking other (E) and distancing from other (F) and their relationships to different moral emotions in both groups. (HC=Healthy Control. MDD = major depressive disorder). Reference category included trials in which participants selected other or no emotion.
